# Inhibition of RANKL and Sema4D improves residual ridge resorption in mice

**DOI:** 10.1038/s41598-022-08016-3

**Published:** 2022-03-08

**Authors:** Meri Hisamoto, Shunsuke Kimura, Kai Iwata, Toshihiko Iwanaga, Atsuro Yokoyama

**Affiliations:** 1 Department of Oral Functional Prosthodontics, Division of Oral Functional Science, Faculty of Dental Medicine, Hokkaido University, Sapporo, 060-8586 Japan; 2 Laboratory of Histology and Cytology, Graduate School of Medicine, Hokkaido University, Sapporo, 060-8638 Japan; 3 Division of Biochemistry, Faculty of Pharmacy, Keio University, Tokyo, 105-8512 Japan

**Keywords:** Diseases, Endocrinology

## Abstract

Residual ridge resorption (RRR) is a chronic and progressive bone resorption following tooth loss. It causes deterioration of the oral environments and leads to the pathogenesis of various systemic diseases. However, the molecular mechanisms and risk factors for RRR progression are still unclear and controversial. In this study, we developed a tooth extraction model using mice for analyzing long-term morphological and gene expression changes in the alveolar bone. We further applied ovariectomy to this model to elucidate the effects of osteoporosis on RRR progression. As a result, the alveolar bone loss was biphasic and consisted of rapid loss in the early stages and subsequently slow and sustained bone loss over a long period. Histological analysis indicated that ovariectomy prolonged the activation of osteoclasts in the alveolar bone. Furthermore, the expressions of *Tnfsf11* and *Sema4d* kept increasing for a long time in OVX mice. Administration of neutralization antibodies for receptor activator of NF-κB ligand (RANKL) effectively suppressed RRR. Similarly, inhibition of Semaphorin 4D (Sema4D) also improved alveolar bone loss. This study demonstrated that reduced ovarian function may be a risk factor for RRR and that RANKL and Sema4D suppression are potential treatments.

## Introduction

Residual ridge resorption (RRR) is a continuous, often lifelong, alveolar bone resorption occurring after tooth loss. Losing teeth in adult is the result of injury or disease, such as dental avulsion, tooth decay, and periodontal disease. The proportion of people who lost their teeth increases with age. In Japan, 40% of people in their late 40 s and 60% of people in their early 50 s have lost at least one tooth^1^. In the United States. 26% of adults aged 65 or older have eight or fewer teeth and about 17% in them have lost all of their teeth^2^. RRR therefore can occur in anyone with a high probability.

RRR follows initial wound healing, which includes epithelial integrity restoration accompanied by bone formation within the extraction socket and bone resorption at the edge of the socket. After the initial rapid healing stage, bone resorption slows down but sometimes persists for a long time. As RRR progresses, dentures become unstable, and dental implant placement becomes difficult, thus worsening the oral health-related quality of life and social activity. Several studies have suggested that prolonged alveolar bone resorption is caused by aging, excessive mechanical stress, and periodontitis^3–5^. However, the risk factors of RRR progression are still controversial.

Osteoporosis is a metabolic disease that affects postmenopausal women. This disease is commonly characterized by low bone mass and bone tissue deterioration, which can lead to increased risk of bone fractures. However, not all individuals with osteoporosis develop RRR. Some animal studies have demonstrated positive correlations between alveolar bone loss or delayed healing of extraction sockets and systemic osteoporosis^6,7^. Contrarily, others have shown a weak relationship or no relevance at all^8,9^. Thus, the relationship between osteoporosis and jawbone resorption has not been elucidated.

The treatment for osteoporosis mainly involves two drug types. Most of the currently available drugs for osteoporosis are bisphosphonates or anti-RANKL monoclonal antibody drugs. These drugs inhibit osteoclastic bone resorption; however, they can sometimes, but not necessarily, induce drug-related osteonecrosis of the jaw (ARONJ) following tooth extraction^10,11^. Another type of drug promoting osteoblastic bone formation is parathyroid hormone (PTH). Intermittent administration of PTH can treat osteoporosis of long bones and vertebrae^12^, and some studies have indicated that intermittent PTH therapy could increase the mineral density of the jawbone in animal models^13,14^. However, intermittent PTH treatment only initially increases bone formation and promotes bone resorption; therefore, the treatment period is limited. A Sost-specific antibody treatment, which promotes bone formation and inhibits bone resorption, was developed and used clinically. An animal study suggested that sclerostin inhibition increased the alveolar bone volume and architecture in rats with alveolar bone loss^15^.

Semaphorin 4D (Sema4D) is expressed by osteoclasts and is a mediator of osteoclast–osteoblast communication. Moreover, it inhibits osteoblastic bone formation. Injection of Sema4D-specific antibodies into ovariectomized mice was shown to promote osteoblastic bone formation without affecting osteoclastic bone resorption in the femur^16^. However, there have only been a few reports on the therapeutic effect of Sema4D inhibition on jawbone loss following tooth extraction.

This study aimed to (1) elucidate the factor of alveolar bone resorption using a murine tooth extraction model and (2) investigate whether injection of RANKL-specific antibodies or Sema4D-specific antibodies could be a useful approach to the suppression of alveolar bone resorption in an ovariectomized mouse model of postmenopausal osteoporosis.

## Results

### Maxillary teeth extractions in mice does not induce long-term loss of maxillary alveolar bone volume

RRR in humans is characterized by sustained loss of the mandible and maxillary bone mass for prolonged periods following tooth extraction. However, there is little information about bone morphological changes after extraction in other mammals, including rodents. Therefore, we investigated the morphological changes of the maxillary alveolar bones of mice following teeth extractions using µCT imaging. At 16 weeks after teeth extractions, the maxillary bone was absorbed on the maxillary sinus side and the maxilla alveolar crest side but was only absorbed slightly on the buccal side (Supplementary Fig. S1 online).

**Figure 1:**
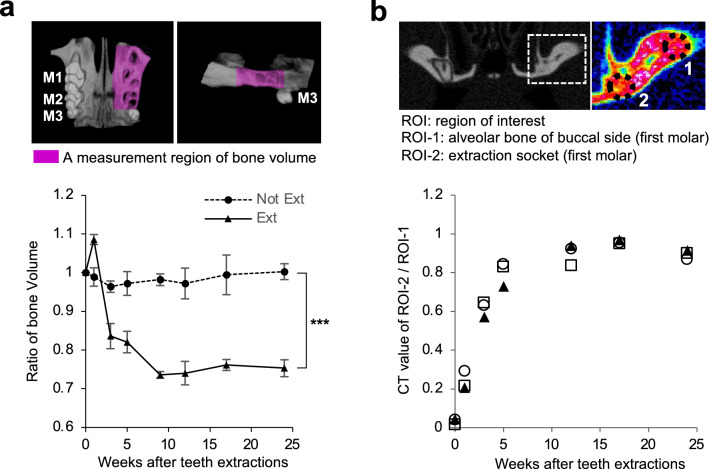
Resorption of the maxillary alveolar bone after teeth extractions in mice. (**a**) Bone volumes of both the extracted and non-extracted sides were determined with μCT analysis. The measurement range of bone volume is indicated in purple in the µCT-3D image of the upper images, which is between the M1 mesial buccal root and M2 distal buccal root. M1, M2, and M3 denote the first molar, second molar, and third molar, respectively. The graph in the lower panel shows the time-dependent changes in bone volume of the teeth-extracted and the non-extracted sides and are expressed as a ratio of the bone volume on day 0 after tooth extraction in the same individual. The data are expressed as mean ± S.D. (n = 3) and are representative of three independent experiments. (**b**) The selected regions of interest (ROIs) in or areas near the extraction socket of the first molar are displayed in the circled areas of the µCT image in the upper panel. The graph in the lower panel shows the time-dependent changes in the ratio of the CT values of the maxilla alveolar bone of the extracted region (ROI-2) to the non-extracted buccal side (ROI-1). Each symbol represents an individual mouse. The data shown in the graphs are representative of three independent experiments. ***P < 0.005; calculated using two-way ANOVA.

We examined the long-period changes of BV on the extracted and non-extracted sides of the maxillary bone from day 0 to 24 weeks following teeth extractions. The BV of the extracted side rapidly decreased to 73.6% ± 1.51% (mean ± standard deviation) by 9 weeks post-extraction (Fig. 1a). Subsequently, although the alveolar BV slightly decreased, there was no significant difference between 9 and 24 weeks (*P* = 0.27 calculated using Student’s *t*-test). This result suggested that alveolar bone resorption following teeth extractions is rarely long-lasting in healthy mice.

The CT value at an extraction socket of the first molar recovered to 80.0% ± 0.06% of the amount of the adjacent alveolar bone of the buccal side at 5 weeks post-extraction, indicating that the extraction socket of the first molar was almost filled with new bone by 5 weeks post-extraction (Fig. 1b).

These results indicated that the sharp decrease in alveolar BV in the weeks following teeth extractions was related to the activation of bone remodeling during healing of the extraction socket^17,18^.

### Ovariectomy promotes long-term resorption of the maxillary bone after teeth extractions

**Figure 2:**
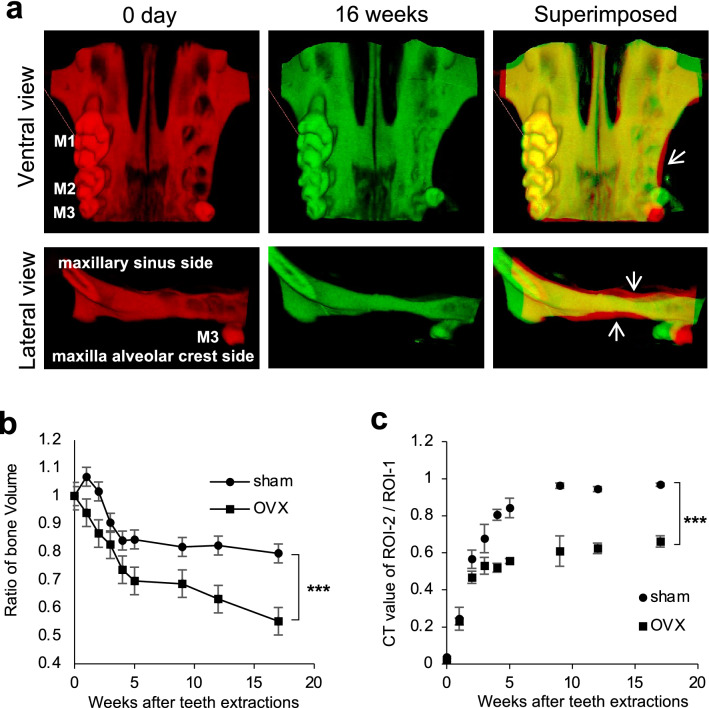
Ovariectomy prolongs maxillary bone resorption after teeth extractions. (**a**) Ventral views (upper images) and lateral views (lower images) of μCT-3D images of the maxilla at day 0 (red) and 16 weeks (green) post-extraction of OVX mice are shown in the left and the middle images, respectively. The right image shows a superimposed illustration of day 0 and 16 weeks post-extraction. The arrows indicate decreased regions of the alveolar bone after 16 weeks post-extraction. (**b**) The graph shows the time-dependent changes in the bone volumes of the teeth-extracted side of the alveolar bone as described in Fig. 1a. Closed squares represent OVX mice and closed circles represent sham mice as the control. Data are expressed as mean ± S.D. (n = 3). (**c**) The graph shows the time-dependent changes in the ratio of the CT values of the maxilla alveolar bone of the extracted region (ROI-2) to the non-extracted buccal side (ROI-1) of the alveolar bone of OVX mice (closed square) and sham mice (closed circle), as described in Fig. 1b. The data shown in the graphs are representative of two independent experiments. ***P < 0.005; calculated using two-way ANOVA.

Postmenopausal osteoporosis could be one of the possible risk factors for ridge resorption^19^. We analyzed bone morphology following teeth extractions using OVX mice as an animal model for osteoporosis. The images obtained via superimposition of CT-3D images revealed that the height and width of the alveolar bone were reduced after 16 weeks post-extraction in OVX mice (Fig. 2a).

The temporal observation of alveolar BV with µCT indicated that the BV in OVX mice rapidly decreased to 69.7% ± 2.60% at 5 weeks post-extraction and subsequently decreased gradually to 55.3% ± 2.63% by 17 weeks following teeth extractions. This result indicated that alveolar bone resorption in OVX mice continues over the long term. Meanwhile, the BV in the sham mice decreased to 84.5% ± 0.30% at 5 weeks and then slightly decreased, but was not statistically significant (*P* = 0.267 calculated using Student’s *t*-test), to 79.5% ± 0.49% at 17 weeks (Fig. 2b). This kinetic BV change in the sham mice was almost identical to the non-treated mice (Fig. 1a).

The ratio of the CT values in the extraction socket of sham mice recovered to 84.2% ± 3.05% at 5 weeks after extraction and reached 96.7% ± 0.89% at 17 weeks, whereas the CT value in that of OVX mice only recovered to 55.5% ± 1.33% at five weeks and subsequently remained almost unchanged during our observation period up to 17 weeks (66.1% ± 3.06%) following teeth extractions (Fig. 2c). This result indicated that the extraction socket was not entirely restored in the OVX mice.

### Prolonged activation of bone remodeling during the restoration of extraction sockets in OVX mice

**Figure 3:**
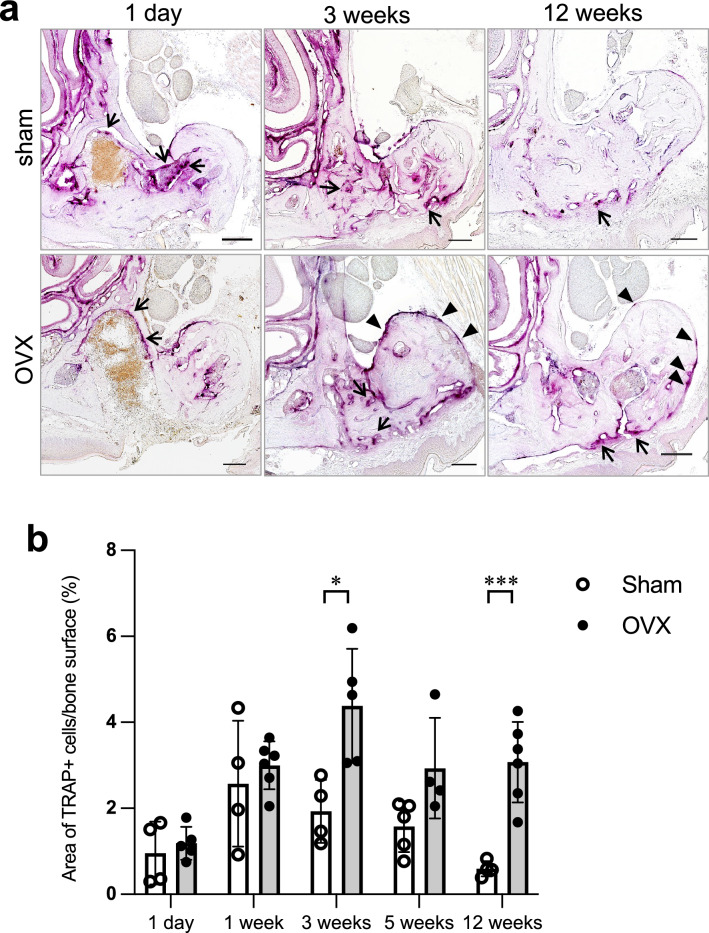
Prolonged increase of osteoclasts in the maxillary bone of OVX mice. (**a**) Enzymatic histochemistry staining for TRAP (red) on the decalcified maxillary alveolar bone at day 1, 3 weeks, and 12 weeks of sham or OVX mice following teeth extractions. The arrows indicate TRAP-positive signals inside and around the extraction socket. The arrowheads indicate TRAP-positive signals on the buccal side of the maxillary alveolar bone. Bars: 100 μm. (**b**) The graph shows the time-dependent changes in the ratio of the total area of TRAP-positive cells to the surface area of the maxilla alveolar bone of the extracted side in sham or OVX mice. The bone of the extracted side was around M2. Data are expressed as mean ± S.D. (sham mice: n = 4–5. OVX mice: n = 4–6), *P < 0.05, ***P < 0.005; calculated using calculated using Student’s *t*-test.

We examined the temporal distribution of osteoclasts during healing of the extraction sockets using enzymatic histochemistry of TRAP, which demonstrated that osteoclasts increased around the extraction sockets immediately after teeth extractions (Fig. 3a). High TRAP activity persisted in the tooth extraction area, especially on the surface of the buccal side of the alveolar bone of OVX mice even at 12 weeks following teeth extractions. Quantitative image analysis revealed that there was prolonged activation of osteoclasts around the tooth extraction site of OVX mice (Fig. 3b).

**Figure 4:**
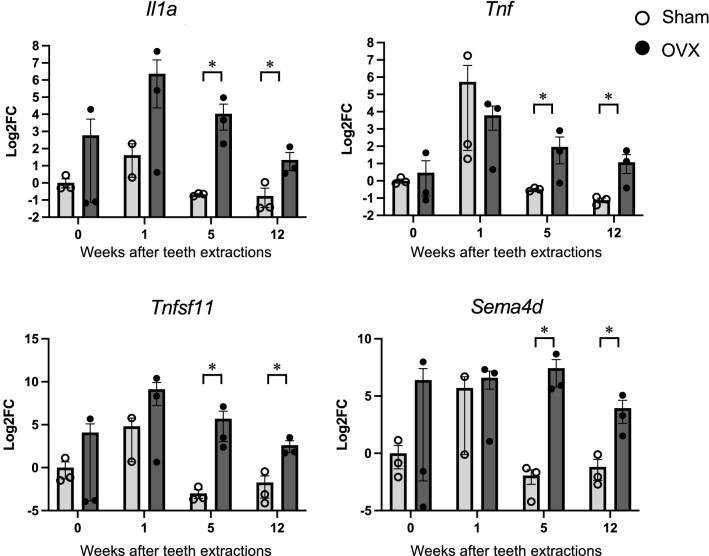
Ovariectomy sustains prolonged high expression of several mRNAs encoding bone metabolism and proinflammatory cytokines after teeth extractions. The expressions of mRNA encoding bone metabolism in the maximally alveolar bone were examined via quantitative PCR in both OVX mice (closed circle) and sham mice (open circle). The graphs show relative expressions of indicated genes normalized to the expression level of day 0 of the sham mice. Data are expressed as mean ± S.E. Three mice were used in each group. *P < 0.05; calculated using Student’s *t*-test.

Quantitative PCR analysis indicated persistent increases in the expressions of genes encoding proinflammatory cytokines, including *Tnf and Il1a* in OVX mice (Fig. 4 and supplementary Fig. S3 online). Similar increased expressions in OVX mice were observed for genes encoding osteoblast-related molecules, including *Alpl*, *Bglap*, *Tnfsf11*, *Dkk1*, *Slc2a1*, *Sema4d*, *Sost*, and *Runx2*, except for *Tnfrsf11b*, in OVX mice (Fig. 4 and supplementary Fig. S3 online). On the other hands, osteoclast-associated genes were increased at 5 weeks after teeth extractions in OVX mice but at 12 weeks they fell to the same level as the sham mice (Supplementary Fig. S3 online).

### Neutralization of RANKL or Sema4d can prevent bone resorption of alveolar bone

RANKL is expressed on the surface of osteoblasts and enhances osteoclastogenesis^20^. Sema4d derived from osteoclasts inhibits osteoblastic bone formation^16^. Consequently, both molecules may promote bone resorption. We confirmed that the increased expressions of genes *Tnfsf11* and *Sema4d* encoding RANKL and Sema4D, respectively, in alveolar bone were sustained at least until 12 weeks after teeth extractions (Fig. 4).

**Figure 5:**
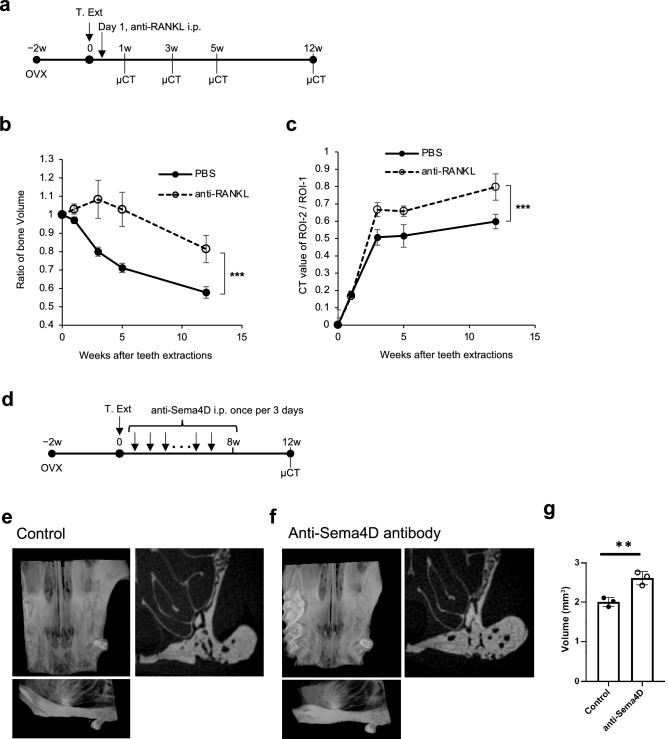
Administration of neutralization antibodies for RANKL or Sema4D can prevent prolonged bone resorption of the maxillary alveolar bone of OVX mice. (**a**) Experimental scheme of administration of the neutralization antibody and µCT observation. T.Ext; teeth extractions. (**b**) The graph shows the time-dependent changes in the bone volumes of the teeth-extracted side of the alveolar bone of OVX mice administrated with anti-RANKL antibody (open circle) or PBS as control (closed circle), as described in Fig. 1a. Data are expressed as mean ± S.D. (n = 3). (**c**) The graph shows the time-dependent changes in the ratio of CT values of the maxillary alveolar bone of OVX mice as described in Fig. 1b. Open circles represent the mice administrated with anti-RANKL antibody and closed circles represent PBS as control (closed circle). The data shown in the graphs are representative of two independent experiments. ***P < 0.005; calculated using two-way ANOVA. (**d**) Experimental scheme of administration of the neutralization antibody and µCT observation. T.Ext; teeth extraction. (**e** and **f**) Ventral views (upper left images) and lateral views (lower images) of μCT-3D images of the maxillary bone, and the µCT images of extraction socket of the first molar (right images) at 12 weeks post-extraction of OVX mice. The images are the alveolar bone of OVX mice administrated with PBS as the control (**e**) and anti-Sema4D antibody (**f**). (**g**) The graphs show maxillary alveolar bone volumes at 12 weeks after teeth extractions of OVX mice administrated with anti-Sema4D antibodies. Controls are the volume of alveolar bone of mice that have had ovariectomy and tooth extraction but have not been administered antibodies. The mice were euthanized at 12 weeks following teeth extractions. The heads were removed and subjected to µCT analysis, and the BV were measured. The data shown in the graphs are representative of two independent experiments. **P < 0.01 (calculated using Student’s *t*-test).

Therefore, we hypothesized that inhibition of RANKL and Sema4D slows the progression of alveolar bone resorption after tooth extraction; then, we conducted experiments to inhibit RANKL and Sema4D with neutralizing antibodies against each. The neutralizing monoclonal antibody against mouse RANKL, clone OYC1, has been reported as an antibody that stabilizes in the body for at least 4 weeks and suppresses bone resorption^21^. We tried a single injection of anti-mouse RANKL-neutralizing monoclonal antibody intraperitoneally after teeth extraction of OVX mice (Fig. 5a). As the result, the administration of this antibody effectively inhibited the reduction of the alveolar bone volume (Fig. 5b). And the bone in the extraction socket also recovered faster by RANKL inhibition (Fig. 5c).

For the inhibition of Sema4D, anti-Sema4D antibody was intraperitoneally injected once every 3 days for 8 weeks as described previous report^16^ (Fig. 5d). And bone volume of the alveolar bone were measured by µCT images at 12 weeks after teeth extractions of OVX mice (Fig. 5e,f). As the results, the inhibition of Sema4D increased the volumes of alveolar bone after teeth extractions; the BV was increased to 130.0% in the mice administered with anti-Sema4D antibodies compared with that of the control experiment (Fig. 5g). These data indicated the possibility that neutralization of RANKL or Sema4D inhibits long-time bone resorption and sustainably increases bone formation in alveolar bone after teeth extractions.

## Discussion

Bone loss in the mandibular or maxillary bones, which lasts long following tooth loss, is characteristic of RRR in humans. In this study, we used mice as an experimental model to observe long-term maxillary BV for up to 24 weeks after teeth extractions. We found that, in healthy mice, a sharp decrease in BV was observed in the short period after teeth extractions, and subsequently, the loss of BV was slight or almost none. Conversely, ovariectomized mice, used as animal model of postmenopausal osteoporosis, had long-lasting gradual loss of BV after a short and sharp decrease. The decrease in BV observed in ovariectomized mice was similar to that during RRR in humans, which shows a biphasic decline, with a sharp reduction in the early stage and a gradual long-lasting decrease thereafter^22^. The long-lasting loss of maxilla BV after teeth extractions may be promoted by risk factors such as osteoporosis but is not observed in healthy conditions.

Postmenopausal osteoporosis leads to micro-architectural deterioration of bone tissue and low bone mineral density^23^. Some clinical studies reported that the RRR increased in osteoporotic edentulous patients^24,25^. However, other studies demonstrated that there was no statistical relationship between edentulous jaw resorption and osteoporosis^26,27^. Thus, the relationship between RRR and osteoporosis is still controversial and has not been sufficiently elucidated. Our experimental study on a novel animal model revealed the possibility that reduced ovarian function exacerbates RRR. However, clarifying the relationship between human postmenopausal osteoporosis and RRR is insufficient in only our murine model. Additional clinical studies are needed in the future.

We focused on Sema4d, which functions as an inhibitor of bone formation by suppressing osteoblast differentiation and modulating osteoblast mobility^28,29^. Sema4d derived from osteoclasts played a role as an inhibitor of bone formation, and mice with targeted deletion of gene encoding Sema4d or its receptor, Plexin-B1, had increased BV. We further demonstrated that administration of a neutralization antibody for Sema4d efficiently prevented femur bone loss in ovariectomized mice^16^. In our experimental model, administration of Sema4d neutralization antibodies into OVX mice significantly suppressed the loss of alveolar BV after teeth extractions. This result suggested that Sema4d is a factor involved in the promotion of bone resorption after tooth extraction and that inhibition of Sema4d may be a useful treatment for RRR. Consistent with our study, a recent study reported the therapeutic effects of small interfering RNA for silencing Sema4d mRNA, which increased the BV over the total volume of the mandibular bone and prevented alveolar bone height loss in an osteoporotic model^30^.

RANKL is a tumor necrosis factor (TNF) cytokine family and functions as a key factor for osteoclast differentiation and activation. Human anti-RANKL neutralizing antibodies are utilized for the treatment of osteoporosis and cancer-induced bone diseases. Administration of RANKL antibodies into OVX mice also suppressed long-lasting alveolar bone resorption after teeth extractions in our experimental model. Noteworthy, this antibody was sufficiently effective with a single dose immediately after tooth extraction.

It has been pointed out that RANKL-neutralizing antibodies have the risk of causing antiresorptive agent-related osteonecrosis of the jaw (ARONJ), which is local osteonecrosis of the maxilla and mandible with no symptoms in other bones^31,32^. In our experimental model, however, no significant signs of maxilla bone necrosis were found. This result indicated that some additional factors, such as chronic inflammation caused by periodontal disease or excessive stimulation from dentures, may be required for the onset of ARONJ in addition to the suppression of osteoclasts by inhibiting RANKL function.

Ovariectomy increases osteoclast formation and the lifespan by producing osteoclastogenic cytokines and stimulating bone resorption, resulting in rapid bone loss. We found transient increases in several osteoclastogenic cytokines after teeth extractions. In healthy mice, these transient increases immediately returned to near their original level. Contrarily, in OVX mice, higher levels of expression of osteoclastogenic cytokines, including *Tnfsf11*, *Sema4d*, *Il1a,* and *Tnf*, were found to persist for more extended periods, at least until 12 weeks after teeth extractions. On the other hand, quantitative PCR analysis could not detect persistent upregulation of osteoclast-related genes. In this study, we used wide region of alveolar bones including extraction sockets as samples for quantitative PCR. As the result, the number of osteoclasts contained may be reduced, and changes in osteoclast-related genes may not have been detected. Nevertheless, enzymatic histochemistry clearly revealed that the activity of osteoclasts persists in the maxilla of OVX mice after teeth extractions. Our data suggest that RRR progression is caused by prolonged osteoclast activation with reduced ovarian function.

With long-term observation of the maxillary bone in this study, we demonstrated that suppression of bone resorption by administration of anti-RANKL or anti-Sema4D antibodies could improve long-lasting alveolar bone resorption following teeth extractions. The mechanism underlying the development of RRR is still unclear. Our experimental model, therefore, will be a good tool for studying ridge resorption and developing therapeutic drugs.

In conclusion, (1) bone resorption after tooth extraction did not progress without a risk factor in our murine model; (2) Reduced ovarian function delayed the healing of extraction sockets and may be a risk factor for RRR; and (3) administration of anti-RANKL antibodies or anti-Sema4d antibodies may be a good therapeutic method to delay bone loss by RRR.

## Materials and methods

### Animals

BALB/cAJcl female mice were used in all experiments and were purchased from the CLEA Japan, Inc. The mice were maintained in conventional conditions under standard condition of 12/12 h of light/dark cycle at temperature 25˚C ± 3˚C and 35% to 60% humidity at the animal facility of Graduate School of Medicine, Hokkaido University. One-week acclimation period was provided before the start of the experiment. All animal experiments followed ARRIVE guidelines and approved by the animal care guidelines for the Care and Use of Laboratory Animals in Hokkaido University Graduate School of Medicine, Japan (approval number: 150139).

### The teeth extractions of mice

The left maxillary first molar (M1) and second molar (M2) were extracted from 7-week-old mice under anesthesia via intraperitoneal injection of an anesthesia cocktail of 0.75 mg/kg medetomidine (Nippon Zenyaku Kogyo Co., Ltd.), 4 mg/kg midazolam (Maruishi Pharmaceutical Co., Ltd.), and 5 mg/kg butorphanol (Meiji Seika Pharma Co., Ltd.; supplementary Fig. S2a online). After the teeth extractions, the mice were awakened via injection of 0.75 mg/kg atipamezole (Nippon Zenyaku Kogyo Co., Ltd.), which is an antagonistic regent for medetomidine. The group of mice without teeth extractions were used as an experimental control. Three independent experiments were performed using three mice in each experimental group. The heads of mice at day 0 to 24 weeks post-extraction were scanned using micro x-ray computed tomography (µCT; Latheta LC-200, HITACHI, Japan) at 24-μm voxel resolution with an energy level of 50 kV under anesthesia (supplementary Fig. S2b online). We weighed the mice daily to confirm that no significant weight loss occurred.

The µCT images were imported into the ImageJ software and then processed into three dimensional images to measure the maxillary alveolar bone volume (BV) and CT value. The BVs were compared with the non-extracted sides. The measurement range of BV was between the M1 mesial buccal root and M2 distal buccal root (Fig. 1a). The CT value of the extraction socket of M1 was compared with the alveolar bone of the buccal side of the same side. The region of interest (ROI)-1 was placed in the maxilla alveolar bone of the buccal side, and ROI-2 was placed in the extraction socket (Fig. 1b). Mice were sacrificed at 24 weeks post-extraction.

### Changes in the morphology of maxillary alveolar bones after teeth extractions in OVX mice

Five-week-old female mice were divided into two groups, sham-operated and ovariectomized (OVX), with each group consisting of three mice. Two independent experiments were performed. Ovariectomies were performed under anesthesia via intraperitoneal injection with a cocktail of 0.75 mg/kg medetomidine, 4 mg/kg midazolam, and 5 mg/kg butorphanol. Ovaries were removed using small bilateral dorsal flank incisions after the hair was shaved. Sham-operated mice received similar incisions under anesthesia without ovary removal and used as an experimental control. The maxillary molars (M1 and M2) of the sham mice and OVX mice were extracted at 2 weeks following surgery. The changes in the maxillary bone by µCT were assessed, and the BV and CT values of the maxillary extraction sockets were measured. We monitored the weight of the mice daily to confirm that no significant weight loss occurred. Mice were sacrificed and maxillary bones were harvested at 16 weeks post-extraction.

### Tissue preparation for enzymatic histochemistry of tartrate-resistant acid phosphatase (TRAP)

OVX and sham-operated mice were euthanized by intraperitoneal injection with an overdose of pentobarbital sodium 1 day, 1 week, 3 weeks, 5 weeks, and 12 weeks after tooth extraction. They were perfused with physiological saline through the heart, followed by 4% paraformaldehyde in 0.1 M phosphate buffer (pH 7.4). The heads of the mice were removed, immersed in the same fixative for 24 h, and decalcified with 5% EDTA for 4 weeks at 4 °C. The decalcified tissues were dipped in 30% sucrose solution overnight at 4 °C, embedded in OCT compound (Sakura Finetek, Tokyo, Japan), and quickly frozen in liquid nitrogen. Frozen sections, about 16 μm in thickness, were mounted on MAS-coated glass slides.

Histological sections were stained with TRAP Staining Kit in accordance with the manufacturer’s protocol (Wako, Japan). The nuclei were stained with hematoxylin. The stained sections were observed and captured by a light microscope with digital camera (BX51 with DP-80, Olympus, Tokyo, Japan). The ratio of the total area of TRAP-positive cells to the surface area of the bone on the extracted side in both sham mice and OVX mice was calculated using the ImageJ software (version 1.53f51, http://imagej.nih.gov/ij).

### Quantitative PCR analysis

For the RNA preparation from the maxillary bone, the tissues were snap-frozen in liquid nitrogen and homogenized in liquid nitrogen using a mortar and pestle to powderize it. TRIzol Reagent (Life Technologies) was added to the homogenized tissues, and the total RNA was purified with a high salt solution (Nippon Gene). First-strand cDNA synthesis was completed using ReverTraAce (TOYOBO). Quantitative PCR reactions were conducted using Rotor-Gene 6000 equipment (Qiagen) or StepOnePLUS™ (Thermo Fisher Scientific K.K.) using KAPA SYBR Green Fast PCR Kit (KAPA Biosystems). Most of the specific primers were designed by Primer Bank^33^ and are presented in Supplementary Table S online.

### Administration of neutralized antibodies into OVX mice

The molars (M1 and M2) were extracted at 2 weeks after ovariectomy. Ovariectomized 7-week-old mice were divided into three groups (n = 3 per group). OVX mice were intraperitoneally administered PBS or 1 mg/kg anti-Sema4D antibody (clone BMA-12, BioLegend) once every 3 days for 8 weeks^16^. The mice were euthanized at 12 weeks following teeth extractions. The heads were removed and subjected to µCT analysis (ScanXmate-A080, COMSCAN TECNO CO., LTD. Yokohama Japan), and the BV were measured. For the administration of anti-RANKL antibody (clone OYC1, ORIENTAL YEAST CO., LTD.), the antibody was intraperitoneally injected with 5 mg/kg only once on the next day following teeth extractions. The µCT images of the mouse heads were taken under anesthesia at 0-, 3-, 5-, and 12-weeks post teeth extractions as described in the paragraph of *teeth extractions of mice*.

### Statistics

Statistical analyses in certain time point were conducted using Student’s *t*-test in Figs. 3b, 4, and 5g. Differences between groups in time-dependent changes were analyzed statistically using two-way analysis of variance (ANOVA) in Figs. 1a,b, 2b,c, 5b,c. All statistical analyses were calculated with Prism software (Prism 9 for macOS).

### Ethical approval

The experimental protocols used in the present study were approved by the animal care guidelines for the Care and Use of Laboratory Animals in Hokkaido University Graduate School of Medicine, Japan (approval number: 150139).

## Supplementary Information

Supplementary Information.
