# Supplementary material for: Inhibition of RANKL and Sema4D improves residual ridge resorption in mice
Source: Sci Rep. 2022 Mar 8;12:4094. doi: 10.1038/s41598-022-08016-3 (PMC8904447; doi:10.1038/s41598-022-08016-3)
Supplement: Supplementary file 1 — Supplementary Information. [file 41598_2022_8016_MOESM1_ESM.pdf]

Supplementary Figures and Table

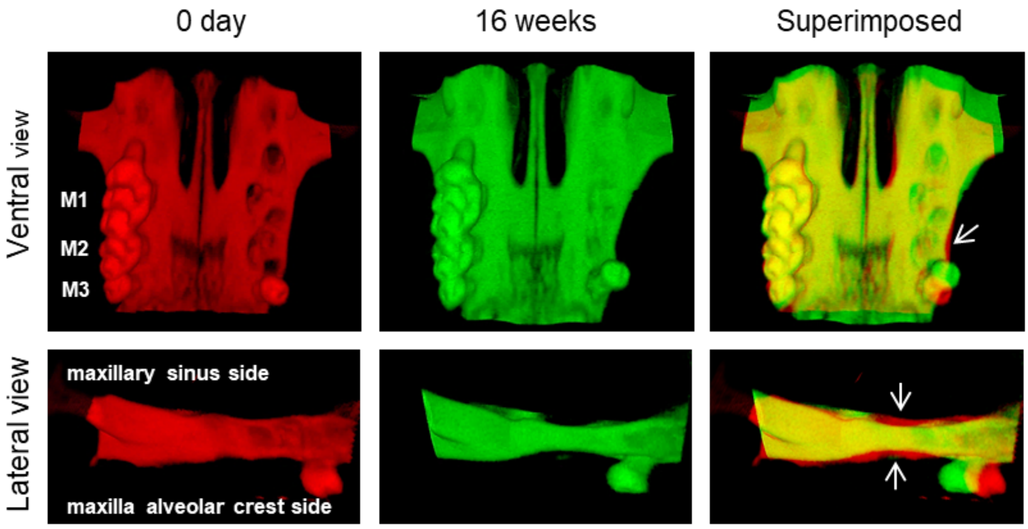

Supplementary Figure S1

The morphological changes of the maxillary alveolar bones of mice following teeth extractions using micro-computed tomography (μCT) imaging. Ventral views (upper images) and lateral views (lower images) of μCT–three dimensional images of the maxilla at day 0 (red) and 16 weeks (green) post-extraction are presented in the left and the middle images, respectively. The right image shows a superimposed illustration of day 0 and 16 weeks post-extraction. The arrows indicate regions with decreased alveolar bone after 16 weeks post-extraction.

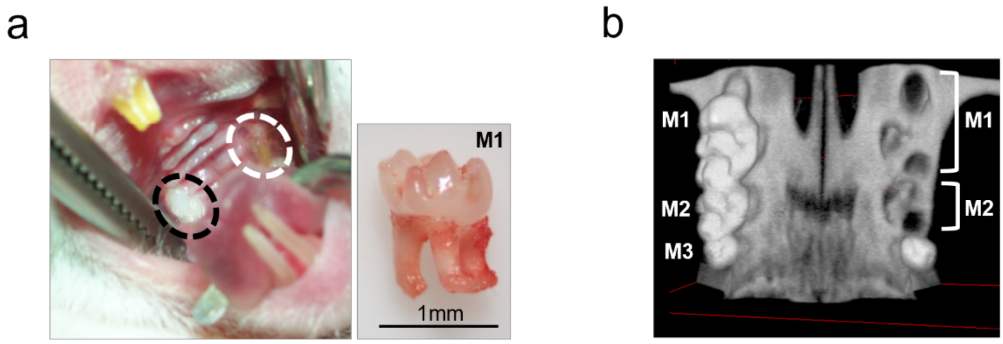

Supplementary Figure S2

The teeth extractions of mice. Stereomicroscopic images (a) of the oral cavity just after tooth extraction and an extracted tooth as well as a μCT image (b) are presented.

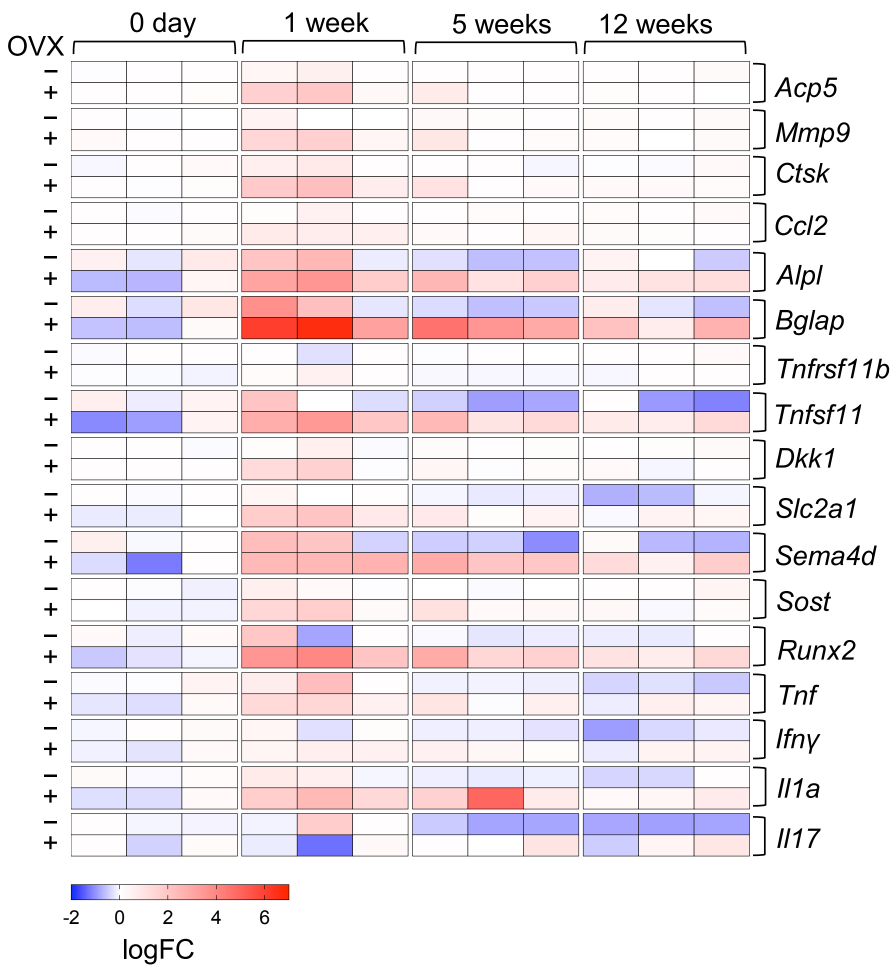

Supplementary Figure S3

Ovariectomy sustains prolonged high expression of several mRNAs encoding bone metabolism and proinflammatory cytokines after teeth extractions. The expressions of mRNA encoding bone metabolism and proinflammatory cytokines in the maximally alveolar bone were examined *via* quantitative PCR in both OVX mice and sham mice. The heatmap shows logFC normalized to the expression level of day 0 of the sham mice. Three mice were used in each group.

	Forward	Reverse	Primer BANK ID
Acp5	CACTCCCACCCTGAGATTTGT	CATCGTCTGCACGGTTCTG	6680624a1
Mmp9	CTGGACAGCCAGACACTAAAG	CTCGCGGCAAGTCTTCAGAG	7305277a1
Ctsk	GAAGAAGACTCACCAGAAGCAG	TCCAGGTTATGGGCAGAGATT	31982433a1
Ccl2	TTAAAAACCTGGATCGGAACCAA	GCATTAGCTTCAGATTTACGGGT	6755430a1
Alpl	CCAACTCTTTTGTGCCAGAGA	GGCTACATTGGTGTTGAGCTTTT	6671533a1
Bglap	CCACACAGCAGCTTGGCCC	AGGCTCCAAGGTAGCGCCGG	
Tnfrsf11b	ACCCAGAAACTGGTCATCAGC	CTGCAATACACACACTCATCACT	31543882a1
Tnfsf11	GACTCCATGAAAACGCAGGT	GAAAGGCTTGTTTCATCCTCC	
Dkk1	CTCATCAATTCCAACGCGATCA	GCCCTCATAGAGAACTCCCG	31542557a1
Glut1 (Slc2a1)	CAGTTCGGCTATAACACTGGTG	GCCCCCGACAGAGAAGATG	22094111a1
Sema4D	CCTGGTGGTAGTGTTGAGAAC	GCAAGGCCGAGTAGTTAAAGAT	7305471a1
Sost	AGCCTTCAGGAATGATGCCAC	CTTTCCCTCCGCATTGACAC	238550154c1
Runx2	AACGATCTGAGATTTGTGGGC	CCTGCGTGGGATTTCTTGGTT	3901266a1
Tnf	CCCTCACACTCAGATCATCTTCT	GCTACGACGTGGGCTACAG	7305585a1
Il1a	CGAAGACTACAGTTCTGCCATT	GACGTTTCAGAGGTTCTCAGAG	52669a1
Ifng	ATGAACGCTACACACTGCATC	CCATCCTTTTGCCAGTTCCTC	33468859a1
Il17a	TTTAACTCCCTTGGCGCAAAA	CTTTCCCTCCGCATTGACAC	6754324a1
Gapdh	TGTGTCCGTCGTGGATCTGA	TTGCTGTTGAAGTCGCAGGAG	

Supplementary Table S: The list of primers used in this experiment.
